# From bedside to bench and back again—molecular mechanisms in acute liver failure

**DOI:** 10.3389/fphys.2014.00018

**Published:** 2014-01-30

**Authors:** Jan-Peter Sowa, Guido Gerken, Ali Canbay

**Affiliations:** Department of Gastroenterology and Hepatology, University Hospital Essen, University Duisburg-EssenEssen, Germany

**Keywords:** acute liver failure, cell death, ethiology, molecular mechanisms, therapeutic options

A major challenge for medical science is the ability to relate findings in cell cultures and animal models back to the patient. In the current setting of technological advance, we now have the ability to over-express or knockdown the expression of specific genes, and we are able to challenge cells with an array of putative factors, but are we able to translate these findings back to the patient? This collection of reviews (which includes unpublished data) aims to summarise our current understanding of acute liver failure (ALF). This has been a daunting task for authors, as ALF is a highly variable condition, whose outcome is determined by a variety of inter-related factors. As such, we are unable to present an exhaustive review of all putative cellular and molecular processes. Nevertherless, all authors have concisely and critically appraised the available literature on the different aspects of ALF, which include ALF pathogenesis, the role of immunity, the diagnostic and treatment strategies, and the role of prognostic algorithms in use today.

ALF can be caused by toxins, infections, metabolic and genetic diseases, but irrespective of etiology, ALF is characterized by the massive and confluent loss of functioning hepatocytes. In their review, Bantel and Schulze-Osthoff (2012) presented putative mechanisms of hepatocyte cell death, and discussed their relevance in patients with ALF. They propose that the degree of hepatocyte cell death may be a surrogate biomarker of ALF severity, and may be utilized as a predictor of ALF outcomes. This is supported findings of etiology dependent modes of hepatocyte cell death in ALF (Bechmann et al., 2010).

While the excessive consumption of alcohol is generally associated with the development of chronic liver disease, the acute intoxication of alcohol can also lead to ALF, or predispose an individual to ALF (from other etiology). It remains unclear how acute alcohol consumption could lead to ALF, although Massey and Arteel (2012) have presented novel data on the possible contributions by PAI-1, fibrins, and integrins.

The importance and role of miRNAs in the development or progression of ALF is only coming to fore. Recent studies show that miRNAs are differentially expressed in liver diseases, and may have a direct pathogenic role in ALF. Elfimova et al. (2012) presented an elegant review of currently identified miRNAs in liver disease, and highlighted the over-expression of miR-122 in patients with acute liver injury. It would be interesting to evaluate if miR-122 could serve as a prognostic biomarker in patients with ALF.

ALF is associated with a massive immune response, with recruitment of inflammatory cells from the peripheral circulation into the liver, the activation of stress and death receptors, and the clearance of apoptotic/necrotic debris, that lead to the perpetuation of hepatic inflammation and injury. In their review, Zimmermann et al. (2012) described the importance of resident macrophages (Kupffer cells) and recruited monocytes in the pathogenesis of ALF. When activated by danger signals, these cells secrete pro-inflammatory cytokines TNFa and upregulate expression of FasL, which in concert, enhance hepatocyte death. Activated immune cells, as well as dying hepatocytes and stromal cells are capable of secreting chemokines that lead to the further recruitment and retention of effector T and NK cells that amplify the inflammatory response. Saiman and Friedman (2012) discussed the role of putative chemokines in ALF, which remains to be fully elucidated as studies have demonstrated apparently conflicting results. This could be related to differences in underlying etiology, and/or to the role of other immune subsets such as regulatory T cells. The use of chemokine inhibitors in treating ALF is attractive, but remains out of reach of clinical applications at this stage, as we have yet to fully understand the roles of specific chemokines and/or immune subsets in the patient.

The liver exhibits a remarkable ability to regenerate itself after an acute insult. For example, after liver resection in man, or partial hepatectomy in rodents, the liver is capable of efficient regeneration that leads to the spontaneous restoration of liver mass and function. During fulminant ALF, however, the loss of hepatocyte mass is just too massive and outweighs the intrinsic ability of residual hepatocytes to regenerate sufficiently. As such, an alternative source of hepatocytes and cholangiocytes is necessary to help restore liver function. Best et al. (2013) reviewed the role of the liver progenitor cell population (LPC) during acute liver injury. They provided examples from preclinical models to show that the LPC is an active participant of liver regeneration, and discussed putative signaling pathways considered important for LPC responses. One such signaling pathway is the Hedgehog pathway that plays an important role tissue development, but has recently been reported to be critical for liver regeneration after partial hepatectomy in mice. Recent data from man confirm that the LPC population is expanded during ALF, and may be useful in predicting the outcome of ALF. Another cell type, which may be involved in ALF are hepatic stellate cell. These could also contribute to liver regeneration by alteration of extracellular matrix (Dechêne et al., 2010).

Whether LPC or stem cells (such as mesenchymal stem cells, MSC or hematopoietic stem cells) may be effective in treating ALF remains to be seen. Hepatocyte transplantation has been successful in rodents, while pre-differentiated MSC have been shown by Christ and Brückner (2012) to ameliorate acetaminophen-induced liver injury. MSC exhibits additional anti-inflammatory properties, thus, may lead to an attenuated inflammatory response. Chamulitrat et al. (2012) suggest a derivative of ursodeoxycholic acid as possible novel treatment option. For the majority of individuals with fulminant ALF, however, a liver transplant (LTx) remains the only curative option. Despite the availability of numerous scoring systems, they are generally poor at predicting survival (i.e., those who do not need a liver transplant). These difficult issues faced by transplant physicians are reviewed extensively by Hadem et al. (2012). Unknown reasons for ALF further complicate clinical handling. Drebber et al. (2013) identified hepatitis E virus as cause for some previously unclear ALF in cases in Europe. Thus, this neglected etiology should also be considered in Europe and not only in Asian or African countries.

In summary, ALF is a challenging disease with many etiologies and mechanisms of injury. A better understanding of the complex pathogenic mechanisms involved is necessary for us to identify new targets for therapy, and/or better predictors of outcome. Studies will be needed to dissect the contributions of individual cell types (recruited vs. resident; parenchymal vs. non-parenchymal) during ALF and regeneration. Future research should focus on novel treatment strategies, including the use of stem cells and LPCs.
